# Variations in internal structure, composition and protein distribution between intra‐ and extra‐articular knee ligaments and tendons

**DOI:** 10.1111/joa.12802

**Published:** 2018-03-02

**Authors:** Yalda A. Kharaz, Elizabeth G. Canty‐Laird, Simon R. Tew, Eithne J. Comerford

**Affiliations:** ^1^ Department of Musculoskeletal Biology Institute of Ageing and Chronic Disease University of Liverpool Liverpool UK; ^2^ The MRC‐Arthritis Research UK Centre for Integrated Research into Musculoskeletal Ageing (CIMA) Liverpool UK; ^3^ Institute of Veterinary Science University of Liverpool Neston UK

**Keywords:** Alcian blue‐periodic acid Schiff, anterior cruciate ligament, extracellular matrix, fascicular matrix, interfascicular matrix, long digital extensor tendon, medial collateral ligament, superficial digital flexor tendon

## Abstract

Tendons and ligaments play key roles in the musculoskeletal system in both man and animals. Both tissues can undergo traumatic injury, age‐related degeneration and chronic disease, causing discomfort, pain and increased susceptibility to wider degenerative joint disease. To date, tendon and ligament ultrastructural biology is relatively under‐studied in healthy, non‐diseased tissues. This information is essential to understand the pathology of these tissues with regard to function‐related injury and to assist with the future development of tissue‐engineered tendon and ligament structures. This study investigated the morphological, compositional and extracellular matrix protein distribution differences between tendons and ligaments around the non‐diseased canine stifle joint. The morphological, structural characteristics of different regions of the periarticular tendons and ligaments (the intra‐articular anterior cruciate ligament, the extra‐articular medial collateral ligament, the positional long digital extensor tendon and energy‐storing superficial digital flexor tendons) were identified using a novel semi‐objective histological scoring analysis and by determining their biochemical composition. Protein distribution of extracellular matrix collagens, proteoglycans and elastic fibre proteins in anterior cruciate ligament and long digital extensor tendon were also determined using immunostaining techniques. The anterior cruciate ligament was found to have significant morphological differences in comparison with the other three tissues, including less compact collagen architecture, differences in cell nuclei phenotype and increased glycosaminoglycan and elastin content. Intra‐ and interobserver differences of histology scoring resulted in an average score 0.7, indicative of good agreement between observers. Statistically significant differences were also found in the extracellular matrix composition in terms of glycosaminoglycan and elastin content, being more prominent in the anterior cruciate ligament than in the other three tissues. A different distribution of several extracellular matrix proteins was also found between long digital extensor tendon and anterior cruciate ligament, with a significantly increased immunostaining of aggrecan and versican in the anterior cruciate ligament. These findings directly relate to the different functions of tendon and ligament and indicate that the intra‐articular anterior cruciate ligament is subjected to more compressive forces, reflecting an adaptive response to normal or increased loads and resulting in different extracellular matrix composition and arrangement to protect the tissue from damage.

## Introduction

Tendons and ligaments (T/Ls) are dense connective tissue that play crucial functions in musculoskeletal system in both humans and animals (Birch et al. 2013). A limited understanding of T/L pathology and an increasing incidence of T/L injuries has led to a major clinical challenge in orthopaedic medicine (Maffulli et al. 2003; Cimino et al. 2010; Kammerlander et al. 2012).

Tendons primarily serve to transfer the forces generated by muscles to the bony skeleton, whereas ligaments serve to connect together different parts of the bony skeleton and passively to stabilise the joint by preventing abnormal joint movement (Benjamin & Ralphs, 1998; Frank, 2004; Screen, 2009; Birch et al. 2013). T/Ls consist of water, cells and an extracellular matrix (ECM). The T/L ECM predominantly comprises collagens (types I, III, V, VI, XII, XIV), with the fibrillar collagen molecules grouped together in a highly ordered fashion, forming fibrils, fibres and fascicles (Kastelic et al. 1978; Clark & Sidles, 1990; Handsfield et al. 2016; Thorpe & Screen, 2016). Fascicles and bundles of fascicles [fascicular matrix (FM)] are surrounded by loose connective tissue referred to as the endotenon/endoligament or interfascicular matrix (IFM), consisting of interfascicular ECM and cells (Clark & Sidles, 1990; Thorpe & Screen, 2016). Besides collagens, T/Ls contain other non‐collagenous extracellular matrix (ECM) components such as proteoglycans and elastic fibres (Frank, 2004; Smith et al. 2011; Thorpe et al. 2013). The precise composition of T/Ls is thought to be related to their specific function and mechanical properties (Mienaltowski & Birk, 2014). Studies have demonstrated that although tendons and ligaments are composed of similar proteins, they contain different proportions of ECM macromolecules. This has been demonstrated in rabbit (Amiel et al. 1984), ovine (Rumian et al. 2007), canine (Kharaz et al. 2016) and human (Little et al. 2014) T/Ls, where altered proportions of molecular components, different collagen organisational structures and protein abundance of some ECM proteins have been demonstrated between the two tissue types. Specialised tendon types such as the energy‐storing superficial digital flexor tendon (SDFT) and positional common digital extensor tendon (CDET) have also been shown to have structural, compositional, proteomic and protein distribution differences which relate to the differing functions of these tendons (Birch et al. 2008; Thorpe et al. 2010, 2012, 2016a,2016b). Ligaments at different locations around the knee joints such as the interarticular anterior cruciate ligament (ACL) and extra‐articular medial collateral ligament (MCL) have been reported to have different collagen content (Fujii et al. 1994), ultrastructural morphometry (Hart et al., 1992) and cellular morphology (Newton et al. 1990). Regional variation of T/Ls can occur as a result of changes in mechanical loading, where regions under mechanical compression can exhibit increased fibrocartilaginous matrix composition (Benjamin & Ralphs, 1998). Such regional variation has also been identified in tendons of other species such as the dog (Okuda et al. 1987), cow (Koob & Vogel, 1987) and rabbit (Daniel & Mills, 1988). Although these studies describe to some extent the compositional and structural differences between T/Ls, none has fully investigated normal non‐diseased T/Ls tissue properties in different anatomical regions.

The canine stifle (knee) joint is highly studied in terms of mammalian musculoskeletal disease due to the high incidence of degenerative joint disease in companion animals such as the dog and as a model for understanding human joint pathology (Proffen et al. 2012). The canine stifle joint is comparable to the human knee joint (Cook et al. 2010) and is similarly predisposed to traumatic injury and non‐contact cranial cruciate ligament (CCL) injury (Comerford et al. 2011), analogous or similar to ACL injuries in man (Serpell et al. 2012). To date, there are few objective data regarding the distinct compositional, structural and morphological characteristics of different T/Ls around the human knee joint and how they are related to ligament and tendon function. Furthermore, differences in the distribution and localisation of ECM macromolecules between ligament and tendon have not been fully explored. This study aimed to use the dog as the animal model for comparison between T/Ls around the stifle joint. We hypothesised that the morphological properties and ECM composition and canine inter‐ and extra‐articular T/Ls around the stifle joint will be different in terms of the location, function and region. We further hypothesised that canine T/Ls around the stifle joint have different ECM macromolecular distributions at the IFM and FM. In this paper we have developed a novel semi‐objective histological scoring analysis to help identify these differential morphological characteristics of T/Ls and have determined T/L differences in the distribution pattern of several ECM proteins using immunostaining.

## Material and methods

### Sample collection and preparation

ACLs, MCLs, long digital extensor tendons (LDET) and SDFTs were harvested following euthanasia from paired (*n* = 5) disease‐free cadaveric canine stifle joints. The stifle joints were from skeletally mature Staffordshire bull terrier dogs (2–5 years old) with a healthy body condition score (4–5/9) (Laflamme, 1997). The dogs were euthanased for purposes not related to this study and ethical approval for use of the cadaveric material was granted by the Veterinary Research Ethics Committee, Institute of Veterinary Science, University of Liverpool (VREC64). The ACL, MCL, LDET and SDFT were divided into three equal sections of proximal, middle and distal regions. In both LDET and SDFT any residual muscle was removed. Further details on the division of the specfic regions of ligaments and tendons for tissue analysis are highlighted in Supporting Information Fig. S1. Further subdivision of each tissue into thirds through each longitudinal section allowed one‐third to be snap‐frozen in liquid nitrogen and stored at −80 °C for biochemical analysis and one‐third to be fixed for 48 h at 4 °C in 4% paraformaldehyde for histological analysis. The remaining third was embedded in a cork disc in Tissue‐TEK OCT (Sakura Finetek; Torrance, CA, USA), snap‐frozen in isopentane and stored at −80 °C until required for analysis.

### Histology

Fixed tissue sections were embedded longitudinally in paraffin wax and 4‐μm sections were cut longitudinally and mounted on polylysine slides. Proximal, middle and distal sections of each tissue were stained with hematoxylin and eosin (H&E), Alcian blue‐periodic acid Schiff (AB‐PAS) stain was used for detection of glycosaminoglycans (GAGs) (Bancroft & Gamble, 2008) and Miller's stain for elastic fibres (Miller, 1971). All histological sections were visualised using a Nikon eclipse 80i microscope and pictures were acquired with a Nikon DS‐L2 standalone control unit.

### Histological scoring and analysis

Histological sections of the ACL, MCL, LDET and SDFT were scored using a three‐part scoring system to assess the cells and extracellular matrix of the tissues (Supporting Information Table S1). All sections were read by two observers (Y.A.K. and E.J.C.) blinded to section location and tissue type on two separate occasions at least 2 weeks apart. The inter‐ and intra‐observer variability was assessed using Kendall's coefficient concordance (Field, 2005).

#### H&E

H&E sections were assessed to determine differences in terms of tissue architecture, cell morphology, cell distribution, vascularisation and inflammation. The scoring system was modified from Stoll et al. (2011), whereby each parameter was numerically graded from 0 to 2 based on changes seen for each parameter listed (Table S1). The average score between inter‐ and intra‐observers was calculated for each parameter.

#### Miller's stain

A modified scoring system from Smith (2010) was used to quantify the differences in term of elastin and microfibril staining. In brief, the increased staining at the IFM and FM, as well as the extent and degree of pericellular staining, was scored based on the degree of the changes [0% = 0 (staining absent), 0–25% = 1 (staining percent in up to 25% of tissue), 25–50% = 2 (staining present in 25–50% of tissue), > 50% = 3 (marked staining in above 50% of the tissue) for each factor. The overall score was added up for each sample, giving a range of possible scores from 0 to 14 (Table S1). These results are referred to as Miller's Score (MS; Smith, 2010).

#### AB‐PAS stain

A similar scoring system to the Miller's score was developed based on Smith (2010) to quantify differences in GAGs staining in the IFM and FM of the T/Ls, as well chondrocytic cell shape changes. The overall score was added up for each sample, giving a range of possible scores from 0 to 14 (Table S1).

### Biochemical analysis for ECM macromolecules

The ECM macromolecular composition of ACL, MCL, LDET and SDFT at the proximal, middle and distal region was determined by measuring total collagen, sulphated glycosaminoglycan (sGAG) and elastin content.

A papain digest was performed to determine the total collagen and sulphated glycosaminoglycan (sGAG) content of ligament and tendon samples. Papain buffer [500 μL; 10 units mL^–1^ papain (P4762, Sigma‐Aldrich, UK) in sterile phosphate‐buffered saline (PBS) with 100 mm sodium acetate, 2.4 mm EDTA and 5 mm cysteine HCL, pH 5.8] was added to the samples (5–20 mg dry weight) which were then incubated for 24 h at 60 °C (Farndale et al. 1986).

Oxalic acid digestion was performed to extract the insoluble elastin from the tissue in the form of soluble cross‐linked polypeptide elastin fragments (α‐elastin). This was done by adding 750 μL of 0.25 m oxalic acid (Sigma‐Aldrich) and by heating samples to 95 °C. Samples were centrifuged at 3000 *g* for 10 min and the supernatant extracted. This process was repeated five times for all tissues to extract all elastin.

The total collagen content was indirectly determined by measuring the imino acid, hydroxyproline (Bergman & Loxley, 1963).

Total sulphated glycosaminoglycan (sGAG) concentrations were measured using the dimethylmethylene blue (DMMB) dye binding assay (Farndale et al. 1986). Elastin content was measured on pooled oxalic acid digested extracts using Fastin dye‐binding assay (Biocolor, UK) (Smith et al. 2014).

### Tissue immunostaining and semiquantitative immunostaining analysis

Distributions of the main ECM components were assessed on the mid‐substance of ACL and LDET (*n* = 3) using immunohistochemistry and immunofluorescence staining for different collagen types, proteoglycans and elastic fibres. The antibodies used were reactive against collagen type I, III, aggrecan, versican, decorin, biglycan, elastin, fibrillin‐1 and fibrillin‐2 (Supporting Information Table S2). All antibodies (apart from elastin, fibrillin‐1 and fibrillin‐2) were used for immunostaining of TLs as described previously (Kharaz et al. 2016), using 4‐μm paraffin‐embedded sections. Frozen sections of 5 μm were used for immunostaining of elastin, fibrillin‐1 and fibrillin‐2 with hyaluronidase (4800 IU mL^–1^ in PBS, H3884, Sigma‐Aldrich) treatment as previously described (Smith et al. 2011). The distribution and arrangement of the selected collagens and proteoglycans were visualised with a Nikon Eclipse 80i. Elastic fibres were assessed with the confocal microscope (Nikon Eclipse Ti). Negative controls were included with rabbit and mouse isotope IgG and normal serum in place of primary antibody. No staining was observed in the control experiments (Supporting Information Fig. S2). Adobe photoshop CS6 software was used to measure the average staining intensity for each antibody stain in each tissue (Zamboulis et al. 2013).

### Statistical analysis

Statistical analysis was performed on biochemical data, histology scoring and semi‐quantitative immunostaining analysis. Normal distribution for each dataset was assessed with graphpad prism (Version 7, GraphPad Prism Software, USA) using a Kolmogorov–Smirnov test. For both biochemical and histological datasets, comparisons between the different locations were performed using one‐way anova with Bonferroni *post‐hoc* test using graphpad prism. An univariate analysis with Bonferroni *post‐hoc* test was also performed using spss (IBM SPSS Statistics, Version 20.0, Chicago, IL, USA) to assess the differences between tissues. Semi‐quantitative immunostaining results were analysed using a *t*‐test in graphpad prism. For all statistical analysis the significance level was set at *P < *0.05. Data are presented as average ± standard deviation.

The integrity of agreement was calculated for intra‐ and inter‐observer concordance between and within both observers, respectively, with Kendall's coefficient using an online software tool (http://www.statstodo.com/KendallW_Pgm.php).

## Results

### Comparison of the morphological characteristics intra‐ and extra‐articular tendons and ligaments

#### ECM organisation

**Figure 1:**
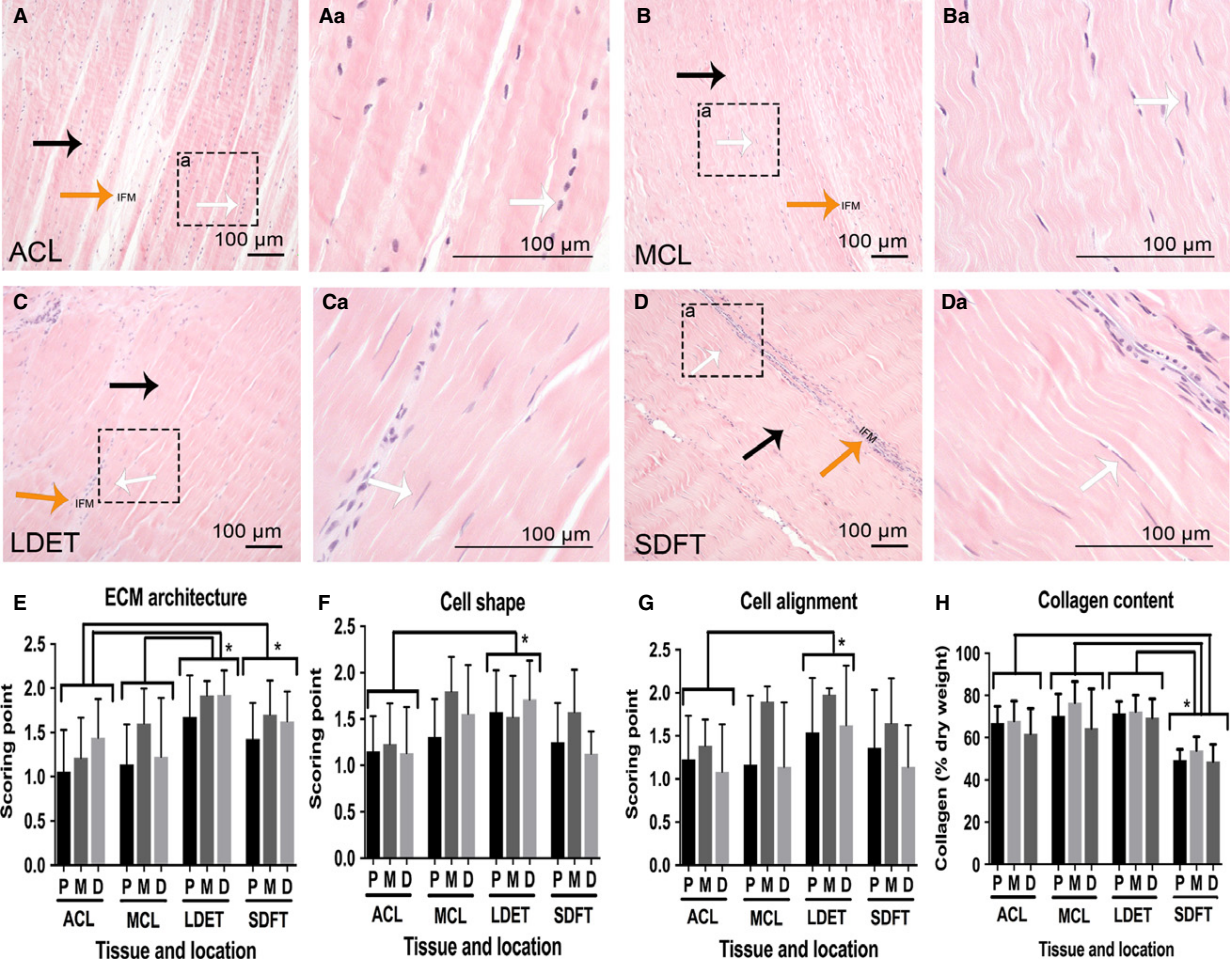
The morphological characteristics and collagen content comparison between intra‐ and extra‐articular tendons and ligaments. Representative H&E staining of anterior cruciate ligament (ACL) (A, Aa), medical collateral ligament (MCL) (B, Ba), long digital extensor tendon (LDET) (C, Ca) and superficial digital flexor tendon (SDFT) (D, Da) middle regions. Histological measurement was performed for proximal (P), middle (M) and distal (D) regions. Boxes and associated letters indicate regions‐of‐interest magnified in the subsequent image. Scale bar: 100 μm. In comparison with ACL and medial collateral ligament (MCL), both the LDET and SDFT were found to have more compact collagen fibre architecture at the fascicular matrix (FM) (black arrows in A, B, C and D and a narrower interfascicular matrix (IFM) (orange arrows in A, B, C and D), which corresponds to the histological scoring of ECM architecture (E). A more heterogeneous population of cell shapes was seen in both ligaments than in either LDET and SDFT, which had more spindle‐shaped cell nuclei (white arrows in A, B, C and D). Histological scoring showed a statistically significant difference in cell nucleus shape between ACL and LDET (F). LDET cells were significantly more uniaxially aligned along the collagen fibres compared with ACL (G). The total collagen content of SDFT was significantly lower than ACL, MCL and LDET (H). No variation was found between different locations in each tissue. Error bars represent SD. *P <* *0.05.

In both the LDET and SDFT the collagen fibres were more compact and aligned in the fascicles containing narrower IFM in comparison with ACL and MCL, resulting in a higher ECM organisation score (Fig. 1A–D). ACL had significantly lower score for ECM organisation compared with LDET (*P *<* *0.001) and SDFT (*P *=* *0.05), which is indicative of a less aligned collagen architecture compared with both tendons. This difference was also observed when MCL was compared with LDET (*P *=* *0.001) (Fig. 1E).

#### Cell shape

In general, a heterogeneous cell nuclei phenotype was seen in all tissue samples with a mixed population of rounded and spindle cell nuclei morphologies (Fig. 1Aa). However, in the ACL, a heterogeneous cell nuclei morphology was observed, which included more rounded and elliptical cell nuclei in the ACL than in the other three tissues (Fig. 1Aa–Da). In the FM of MCL, LDET and SDFT the cell nuclei were more spindle‐shaped and also more elongated in comparison with the ACL (Fig. 1Ba, Ca, Da). This observation was found to be statistically significant between LDET and ACL (*P* < 0.005), as a lower cell shape score was measured for the ACL (Fig. 1F).

#### Cell alignment

Alignment of cells was assessed based on orientation of cells along the collagen fibre bundles. Histological scoring of cell alignment demonstrated significantly higher score in LDET than in MCL (*P *<* *0.05), indicative of a more uniaxial alignment of cells in LDET (Fig. 1G).

#### Cellular distribution

The cellular distribution in the different tendons and ligaments was assessed as normal if cells were not focally increased. Statistical analysis of cellular distribution showed no significant differences between the tissues (*P *>* *0.05) (Supporting Information Fig. S3A).

#### Vascularisation and inflammation

Comparison of both intra‐ and extra‐articular tendons and ligaments for vascularisation and inflammation were assessed based on increased blood vessels and the presence of a cellular infiltrate of cells such as neutrophils, lymphocytes and macrophages. Statistical analysis found significantly more blood vessels and infiltrative cells in the SDFT than in the ACL (*P *<* *0.001), MCL (*P *<* *0.001) or LDET (*P *=* *0.05) (Fig. S3B,C).

No statistically significant differences were found for the histological scoring results between different locations within either tendons or ligaments.

### Inter‐ and intra‐observer agreement histology scoring system

To determine the reproducibility of the newly developed histological scoring procedure, the agreement of scores between different observers or between scores from the same observer taken at least 2 weeks apart was measured. Kendall's coefficient concordance gave an average of 0.71 and 0.64 for observer 1 and observer 2 intra‐observer variations, respectively, and an average value of 0.75 for inter‐observer variations. This indicated a good strength agreement for both intra‐ and inter‐observer scores.

### Tissue distribution and biochemical analysis for ECM macromolecules

#### Collagen content of intra‐ and extra‐articular tendons and ligaments

The average collagen content, as a percentage of dry weight, was 65.6 ± 9.7 in ACL, 70.44 ± 10.8 in MCL, 71.16 ± 11.1 in LDET and 50.8 ± 10.7 in SDFT. The SDFT had statistically significantly less collagen compared with ACL (*P *<* *0.001), MCL (*P *<* *0.001) or LDET (*P *=* *0.001) (Fig. 1H). There were no statistically significant differences in collagen content between the proximal, middle and distal location in each tissue.

#### Glycosaminoglycan distribution and content of intra‐ and extra‐articular tendons and ligaments

**Figure 2:**
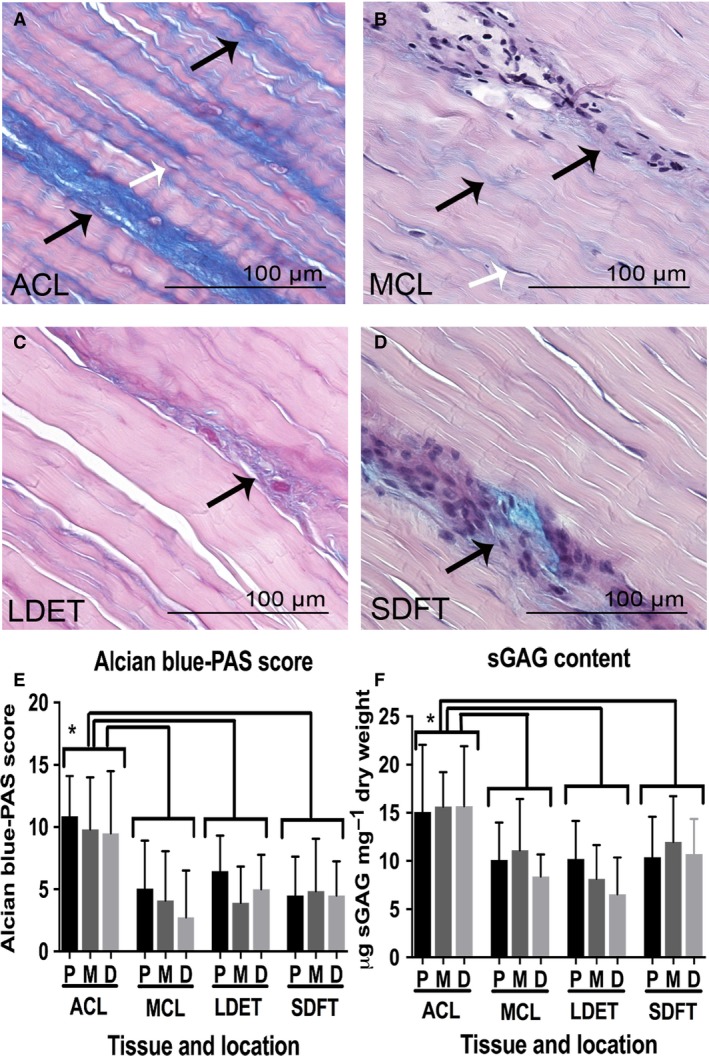
The sulphated glycosaminoglycan (sGAG) distribution and content comparison between intra‐and extra‐articular tendons and ligaments. Representative AB‐PAS staining of anterior cruciate ligament (ACL) (A), medial collateral ligament (B), long digital extensor tendon (LDET) (C) and SDFT (D) middle region. Scale bar: 100 μm. sGAGs were mainly present in the IFM (black arrows in A, B, C and D). Increased staining of sGAGs was particularly observed in ACL with pericellular staining (white arrows in A and B). This finding was statistically significant in both the histological AB‐PAS score (E) and sGAG content measurement (F) and. Error bars represent SD. *P <* *0.05.

sGAGs were distributed mainly at the IFM in both tendons and ligaments. In the ACL, sGAGs had an increased staining subjectively noted at IFM and surrounding the cells in comparison with MCL, LDET and SDFT (Fig. 2A–D). Statistical analysis of the histological scoring for differential sGAG staining between tendons and ligaments, showed a higher AB‐PAS score in the ACL than MCL (*P *<* *0.001), LDET (*P *<* *0.001) or SDFT (*P *<* *0.001) (Fig. 2E). The mean sGAG content as μg mg^–1^ dry weight was 15.5 ± 5.1 in ACL, 9.9 ± 3.9 in MCL, 8.3 ± 3.8 in LDET and 11.1 ± 4.1 in SDFT. The ACL had a statistically greater sGAG content compared with MCL (*P *<* *0.001), LDET (*P *<* *0.001) and SDFT (*P *<* *0.05) (Fig. 2F). There were no statistically significant differences found between proximal, middle and distal locations in both histological AB‐PAS score and sGAG content measurement.

#### Elastic fibre distribution and content of intra‐ and extra‐articular tendons and ligaments

**Figure 3:**
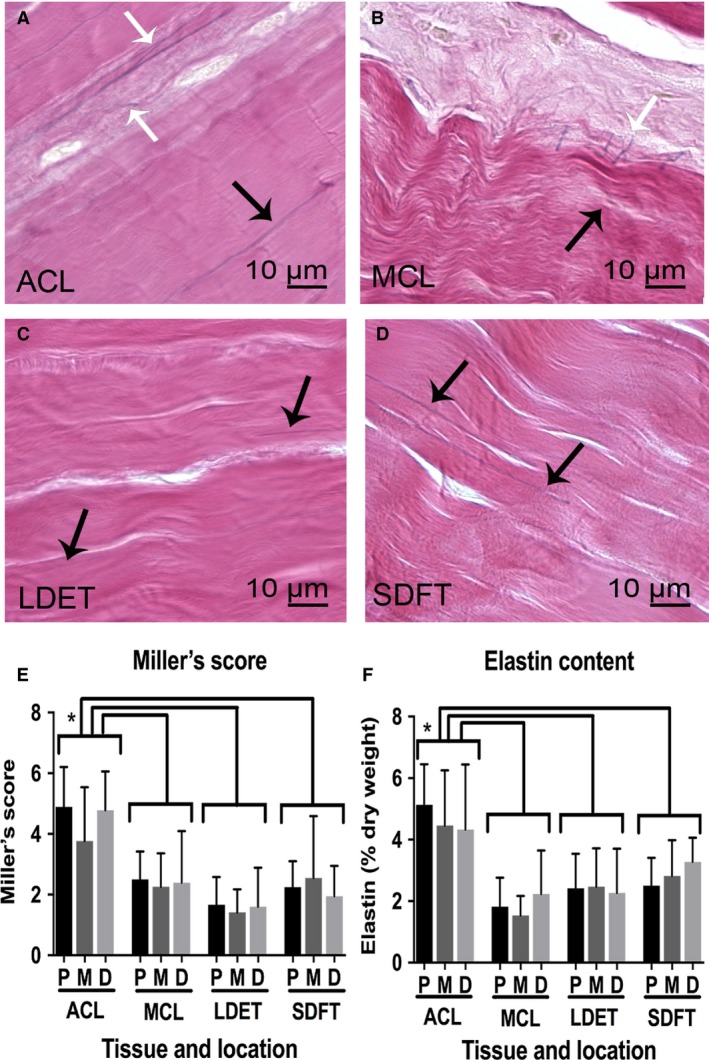
The elastic fibre distribution and content comparison between intra‐ and extra‐articular tendons and ligaments. Representative Miller's staining of anterior cruciate ligament (ACL) (A), MCL (B), long digital extensor tendon (LDET) (C) and SDFT (D) middle region. Scale bar: 100 μm. Elastic fibres were located at the FM (black arrows in A, B, C and D) and IFM (white arrows in A and B). Elastin content was significantly higher in the ACL than in the medial collateral ligament, LDET or SDFT with histological anyalsis using Miller's scoring (E) and by measung the elastin content (F). No variation was found between the different regions in each tissue. Error bars represent SD. *P <* *0.05.

Elastic fibres were mainly located at the IFM but were also found aligned parallel to the collagen fibres and pericellularly (Fig. 3A–D). A further description of arrangement of elastin and the microfibrillar glycoproteins fibrillin‐1 and fibrillin‐2 within the articular and periarticular tendon and ligament tissues is given below. Histological scoring demonstrated more elastic fibres in the ACL than in the MCL (*P *<* *0.001), LDET (*P *<* *0.001) and SDFT (*P *=* *0.001) (Fig. 3E). Elastin content (percentage of dry weight) was 4.6 ± 1.6 in the ACL, 1.9 ± 0.9 in MCL, 2.4 ± 1.1 in LDET and 2.9 ± 0.9 in SDFT. The ACL contained a significantly higher elastin content compared with MCL (*P *<* *0.001), LDET (*P *<* *0.001) or SDFT (*P *<* *0.001) (Fig. 3F). There were no statistically significant differences between proximal, middle and distal regions within the tissues in either the Miller's score and elastin content measurement.

### Distribution of ECM macromolecules in ACL and LDET with immunostaining

#### Collagen type I

**Figure 4:**
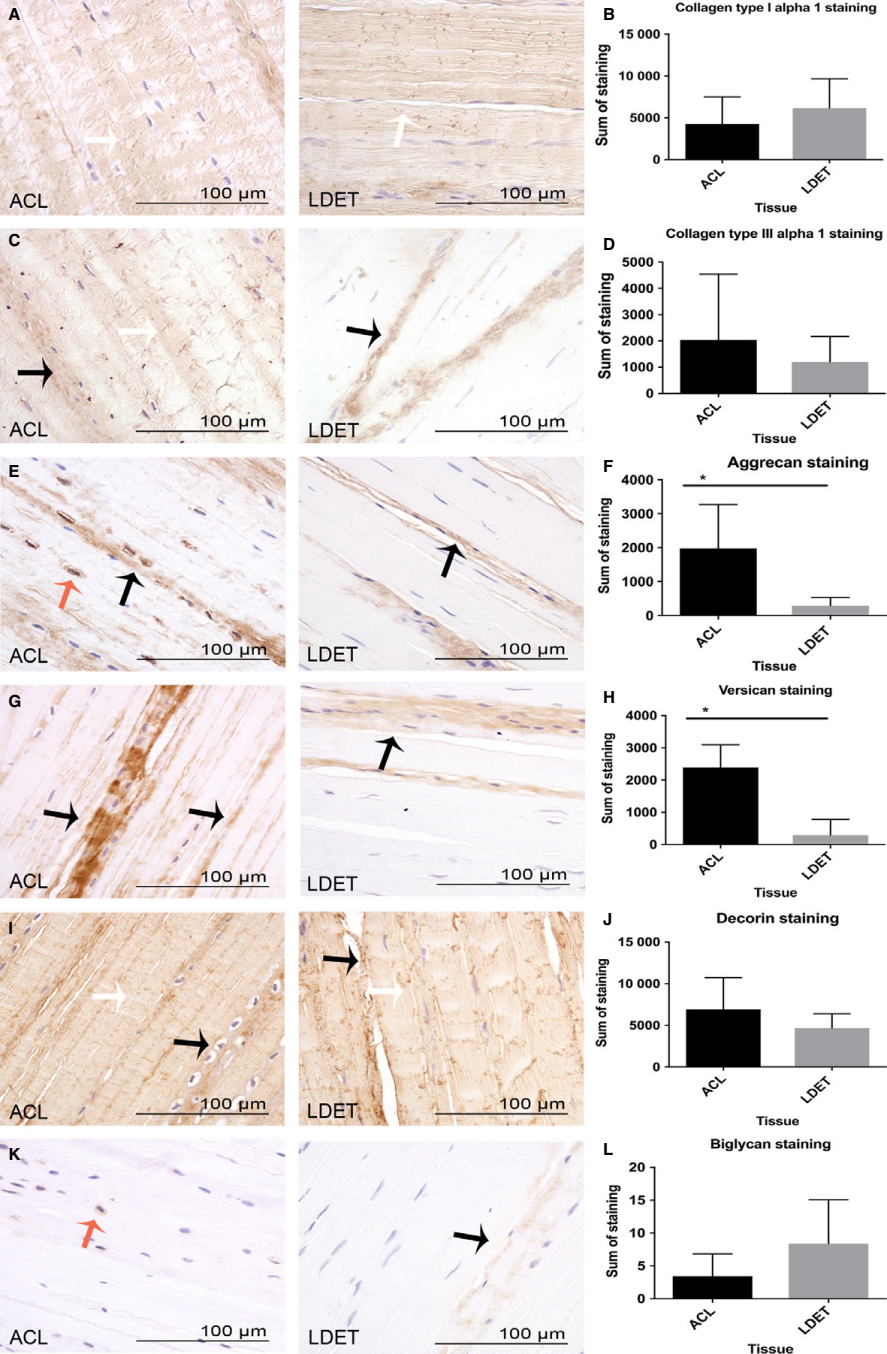
The immunolocalisation of collagens and proteoglycans in articular tendon and ligament. Immunostaining and semiquantitative analysis of collagen type I alpha 1 chain (A and B), type III alpha 1 chain (C and D), aggrecan (E and F), versican (G and H), decorin (I and J) and biglycan (K and L) is demonstrated in the anterior cruciate ligament (ACL) and long digital extensor tendon (LDET). Scale bar: 100 μm. Adobe photoshop CS6 was used to measure greyscale mean intensity and the sum of intensities for all pixels was calculated. The average sum of intensities was measured for each antibody stain in each tissue. Collagen type I immunostaining was mainly present in the aligned fibres (white arrows in A). Collagen type III was present at both the FM (white arrow in C) and IFM (black arrows in C) in ACL, whereas in LDET it was primarily found in the IFM. No significant difference in staining intensity was found for collagen type I or III between the two tissues (B and D). Aggrecan and versican were mainly present at the IFM region in both ACL and LDET (black arrows in E and G). Pericellular staining of aggrecan was also observed in ACL (orange arrow in E). Significantly increased staining intensity of both aggrecan and versican was measured in ACL (F and H). Decorin immunostaining was present in both the FM and IFM in ACL and LDET (white and black arrows in I), whereas biglycan staining intensity was lower and was only occasionally present pericellularly in ACL (orange arrow in K) and at IFM in LDET (black arrow in K). No difference in intensity of staining was measured for decorin and biglyan. Error bars represent SD. *P < 0.05.

In both ACL and LDET the most marked immunostaining for collagen type I was found in the FM, but it was also seen in the IFM (Fig. 4A). There were no significant differences in collagen type I staining between ACL and LDET (Fig. 4B).

#### Collagen type III

In the ACL, collagen type III was found to be present in the FM and IFM, whereas in LDET it was mainly present in the IFM (Fig. 4C), as described previously (Kharaz et al. 2016). There were no statistically significant differences in the immunostaining intensity for collagen type III in ACL and LDET (Fig. 4D).

#### Aggrecan

Marked immunostaining of aggrecan was observed in the IFM regions of ACL compared with LDET (Fig. 4E). Aggrecan was also highly localised around the ligamentocytes (Fig. 4E). There was significantly greater staining of aggrecan in ACL than in LDET (*P *<* *0.05) (Fig. 4F).

#### Versican

Versican was present in ACL and LDET in both the IFM and FM (Fig. 4G). A noticeable immunostaining of versican was noted in ACL in comparison with LDET, and was statistically significantly higher (*P *<* *0.01) (Fig. 4H).

#### Decorin

Immunostaining of decorin was present in the FM and IFM in both ACL and LDET (Fig. 4I). There were no significant differences in decorin intensity staining between the ACL and LDET (Fig. 4J).

#### Biglycan

A minor immunoreactivity of biglycan was present in the IFM of LDET. However, in the ACL, biglycan was only found occasionally surrounding rounded cells (Fig. 4K). There were no significant differences in the intensity of the immunostaining of biglycan between ACL and LDET (Fig. 4L).

#### Elastin fibres and co‐localisation with fibrillin‐1 and ‐2

**Figure 5:**
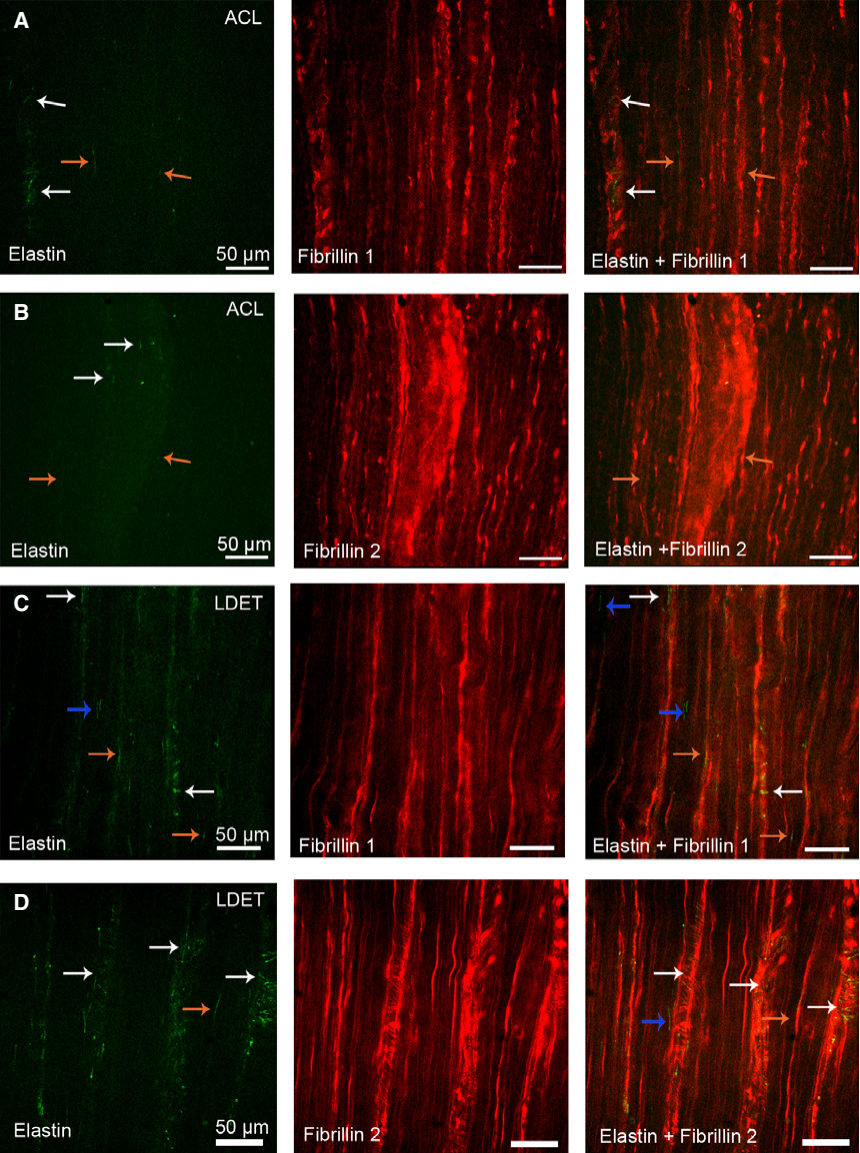
The co‐localisation and distribution of elastic fibres in articular tendon and ligaments. Immunostaining of elastin fibres with fibrillin‐1 and ‐2 in the anterior cruciate ligament (ACL) (A and B) and long digital extensor tendon (LDET) (C and D). Scale bar: 50 μm. Fibrillin‐1 and fibrillin‐2 (red) were found to be localised between collagen fascicles and bundles with a parallel alignment with the long axis of the tissue mainly surrounding the ligament and tendon cells. Elastin (green) was sparse in comparison with fibrillin‐1 and fibrillin‐2. In both ACL and LDET, elastin fibres were mainly distributed in the IFM regions and co‐localised with fibrillin‐1 and ‐2 in this region (white arrows A, B, C and D). Elastin fibres were also found within the ACL and LDET mostly aligned with the fascicles and co‐localised to fibrillin‐1 and ‐2 (orange arrows and A, B, C, D). Occasionally in LDET, not all elastin fibres were found to co‐localise with fibrillin‐1 and ‐2 (blue arrows in C and D).

In both ACL and LDET a similar pattern of distribution of fibrillin‐1 and ‐2 was observed. Immunostaining of both fibrillin‐1 and ‐2 was found to be broadly orientated parallel to collagen bundles with pericellular staining and was more marked in the IFM in both ACL and LDET (Fig. 5A–D, red stain on fibrillin‐1 and ‐2 images). In contrast, elastin fibres were sparse in comparison with the fibrillin‐1 and ‐2 in both T/Ls (Fig. 5A–D, green stain of elastin images). In both ACL and LDET, elastin fibres were found to be predominantly present in the IFM and were arranged in a fine, twisting meshwork either parallel or peripendicular to the long axis of the tissue (Fig. 5A–D, white arrows on elastin images). All elastin fibres in this region were co‐localised with either fibrillin‐1 or 2 in both ACL and LDET (Fig. 5A–D, white arrow on elastin + fibrillin‐1 and fibrillin‐2 images). Elastin fibres were also found in the ACL and LDET FM, where they were mostly oriented parallel to collagen bundles (Fig. 5A–D orange arrows in elastin images). In this region elastin fibres were commonly co‐localised with both fibrillin‐1 and fibrillin‐2, where they were in close proximity to the cells (Fig. 5A–D orange arrows elastin + fibrillin‐1 and ‐2 images). In the LDET, it was occasionally noted that fibrillin‐1 and ‐2 were independent, as elastin was occasionally not found to co‐localise with fibrillin‐1 or fibrillin‐2 (Fig. 5C–D, blue arrows in elastin + fibrillin‐1 and ‐2 images).

## Discussion

This study has demonstrated the different compositional and morphological characteristics between T/Ls around the canine knee joint. We have determined these differences for the first time using both objective scoring systems and biochemical assays. Significantly less compact collagen architecture, more mixed cell morphology as well as the increased presence of GAGs and elastic fibres was found in the ACL compared with the other articular and periarticular T/L tissues. However, no significant regional differences within each tissue were found. A differential distribution of several ECM macromolecules such as aggrecan, versican and elastic fibres between T/Ls was observed, supporting our hypothesis that mammalian T/Ls have distinct distributions of ECM macromolecules likely related to their function.

Analysis of histological sections of ligaments (MCL, ACL) showed that the ACL contained less compact collagen fibres and larger IFM, whereas both LDET and SDFT consisted of more parallel and compact collagen fibres. This difference may be explained by the multiaxial loading pattern in ligaments (Young et al. 2002), resulting in a more complex compositional architecture with regard to the matrix in canine ACL.

Ligaments such as the ACL were also found to have a more mixed population of cell morphologies than LDET. This finding supports the variation in cell morphology previously reported within the canine cruciate ligament complex (Smith et al. 2012). We also found that the cell nuclei of tenocytes in the canine tendon were more spindle‐shaped, which corresponds with observations in tendons from different species including the horse (Clegg et al. 2007) and rabbit (Amiel et al. 1984). Similarly, Amiel et al. (1984) found that rabbit ACL contained more round and ovoid cells when compared with MCL, patellar and Achilles tendon. Murray et al. (2004) reported a majority of fusiform and rounded cell nuclei in normal human ACL which were also found in the canine ACL using histological analysis, indicating similar intrinsic properties of the fibroblasts between two species. The rounded and ovoid cell phenotype in tendon and ligament becomes more prominent close to the bone (origin and insertion regions) in ligament (Duthon et al. 2006) and at the osteotendinous junction in tendon (Docking et al. 2013), likely to be as a result of compressive forces. We found that canine ACL ligamentocytes throughout the different regions and not solely at insertion region had a more epiliptical and rounded cell nuclei phenotype, with cells being mostly surrounded by sGAGs as stained with Alcian blue‐PAS, suggesting a ‘chondrocytic’ appearance. The apparent chondrocytic cell phenotype in the canine ACL agrees with previously reported findings in the ligaments of dogs at low and high risk of ACL rupture (Comerford et al. 2006). Given that in the current study healthy, non‐aged T/Ls tissues were used, our findings in the canine ACL may be a normal discovery and may be as result of physical adaptation, rather than pathological degeneration as reported in human ACL (Hasegawa et al. 2012).

Another important finding of the novel histological scoring system used in this study was the significantly increased sGAG and elastin content found in the ACL compared with the other three tissues examined. Interestingly, these macromolecules were primarily localised within the IFM. This finding was also supported by the increased sGAG and elastin content found in ACL in comparison with MCL, LDET and SDFT measured through biochemical analysis. These results support previous findings in a T/Ls comparison study in sheep (Rumian et al. 2007) and rabbit (Amiel et al. 1984), where higher GAG content was found in cruciate ligaments than in extra‐articular collateral ligaments and several tendons. The increased proteoglycan content in ACL may allow for more slippage and lubrication between collagen fibrils and fibres, allowing a greater degree of deformation to prevent damage during sports‐related activities (Rumian et al. 2007). In the equine tendon the capacity for fascicle sliding has been demonstrated to be different between the energy‐storing equine SDFT and positional CDET, which is the result of interfascicular differences (Thorpe et al. 2012). In the SDFT, the IFM has been reported to withstand more cyclic loading and is more elastic than the CDET (Thorpe et al. 2015). The greater degree of deformation in the ACL may also be reflected by the increased elastin content that was measured compared with the other three tissues examined. Elastin has been reported to contribute to the mechanics of ligaments, primarily in the toe regions of the stress–strain curve of porcine MCL, thus contributing to its viscoelastic properties (Henninger et al. 2013, 2015). Together these data imply that the increased proteoglycan and elastin content in the IFM of the ACL may lead to an increase in elastic and viscoelastic properties of this tissue. Nevertheless, the role and function of the IFM in ligaments has yet to be established. Our findings may also be related to the more specialised mechanical function of the ACL compared with other tissues, as a previous study has shown differences in material strength and stiffness between equine suspensory ligament, and SDFT and CDET (Birch et al. 2013).

In the current study, the regional variances of level of matrix constistuents in tendon and ligaments were also assessed; however, we found no statistically significant differences between these locations in any histological or biochemical measurements. This may be explained by the small proportion of this region which has been examined for the various analyses and may therefore mask localised differences between the regions. Future studies will include the use of laser capture microdissection to obtain a precise separation of the different regions of tendons and ligaments.

This study also aimed to assess the distribution of the ECM macromolecules between tendon and ligament. Both ACL and LDET were primarily chosen based on the findings of different ECM compositions within these tissues and morphological and/or structural differences (as discussed above) in the ACL. In comparison with the ACL, the LDET was found to differ in terms of ECM composition, structure and cellular morphology. As this positional tendon is also located within the canine stifle joint it was considered to be more comparable to the ACL. The distribution and organisation of ECM macromolecules were assessed in the middle region of the tissues, as no regional differences in biochemical composition were found, but also to avoid any potential fibrocartilaginous origin or insertional regions in both tissues. ECM proteins, including collagen type I, III, aggrecan, versican, decorin and biglycan, elastin, fibrillin‐1 and ‐2, were analysed using immunohistochemical staining. Collagen type I immunostaining was found to be intense and mainly present in the FM in both LDET and ACL. Collagen type III was primarily located at IFM regions in LDET, similar to that previously reported for normal equine SDFT (Sodersten et al. 2013) and human extensor carpi radialis brevis tendon (Duance et al. 1977). However, we found that collagen type III was not only located at the IFM but was also aligned throughout the fibre bundles in the ACL. We did not find the increased immunostaining of collagen type III in ACL to be statistically different to that of the LDET; however, we have previously reported, using mass spectrometry, an increased abundance of collagen type III in ACL compared with the LDET (Kharaz et al. 2016). This difference could be due to the fact that mass spectrometry is more sensitive than immunohistochemical techniques, which includes the ability to detect small differences in protein levels between samples (Little et al. 2014). In the current study, the widespread distribution of collagen type III located throughout the ACL might indicate that in the ligament, collagen type III plays more of an essential role in bridging collagens with adjacent matrix, which could be important for the pliability of the ligament; however, this needs to be elucidated further. The intensity of decorin staining in both ACL and LDET was found to be similar and was found to be distributed at both FM and IFM, indicative of binding to collagen types I and III. In contrast to decorin, biglycan immunostaining was present in LDET IFM and occasionally pericellularly in ACL. This finding supports studies where low mRNA expression and immunostaining of biglycan was observed in the canine ACL (Yang et al. 2012). In contrast to biglycan, increased immunostaining of both aggrecan and versican was found in ACL compared with LDET. Both aggrecan and versican were localised mainly in the IFM of ACL and LDET; however, aggrecan was also found to be located pericellularly only in ACL. This agrees with our previously reported mass spectrometry results of canine ACL and LDET, where an increased protein abundance of aggrecan was found in ACL in comparison with LDET (Kharaz et al. 2016). The increased immunostaining of aggrecan and versican at the ACL mid‐region as compared with tendon indicates that the canine ACL might also undergo compression at the central region where it is twisted around the posterior cruciate ligament under tensile strength (Comerford et al. 2006). Therefore, the ACL appears to have a different ECM composition and arrangement, possibly to protect the tissue from damage and to better withstand compression.

The distribution of elastin, fibrillin‐1 and fibrillin‐2 was assessed to determine whether T/Ls from the same species and breed have a different or similar distribution of elastic fibres. Fibrillin‐1 and ‐2 were found to be aligned along the long axis of the tissue and surrounding ligament and tendon cells. An increased intensity of staining was also observed in the IFM. The similar distribution of both fibrillins may indicate co‐localisation of both fibrillin‐1 and fibrillin‐2, as has been shown previously in bovine tendon (Grant et al. 2013). In comparison with fibrillin‐1 and fibrillin‐2, elastin fibres were sparse and were located more at the IFM, but the fibres were also found in the ACL and LDET FM. This has also been recently reported by Godinho et al. (2017) who measured increased elastin equine SDFT IFM, which is suggested to play an important function in the elastic recoil ability of the energy‐storing SDFT IFM. In the current study, elastin was present either in between collagen fibre bundles or orientated along the fibres; it was found to co‐localise with both fibrillin‐1 and fibrillin‐2, where it was also in close proximity to both ACL and LDET cells. These findings support the previously demonstrated elastic fibre distribution in bovine tendon (Grant et al. 2013) but the distribution was slightly different to that previously reported for canine ACL, where elastin was found to co‐localise with fibrillin‐2 but not fibrillin‐1 (Smith, 2010). This may be due to breed differences, as this study was conducted in ACLs from ex‐racing greyhounds. The distribution of elastic fibres in the IFM may provide elastic recoil and offer stress protection of blood vessels and nerves in this region (Grant et al. 2013; Godinho et al. 2017) and play an important role in the microenvironment of both LDET and ACL cells.

In conclusion, our study supports the hypothesis of differences in ECM morphology, composition and protein distribution among canine intra‐articular ACL, extra‐articular MCL, positional LDET and energy‐storing SDFT. This study is the first to use a histological scoring system for semi‐quantitative analysis of morphological and structural differences between ACL and other T/L tissues. Notable morphological differences include less compact collagen architecture, differences in the shape of cell nuclei, and increased GAG and elastin content in the ACL than in the other three tissues. The localisation of collagen type III, aggrecan and versican were found to differ between ligament (ACL) and LDET tendon (LDET). The increased intensity of aggrecan and versican may increase the hydration and viscoelastic properties of the ACL, contributing to the very specialised joint function of this tissue. Differences in the distribution and arrangement of ECM collagens, proteoglycans and elastic fibres between FM and IFM in both LDET and ACL are suggestive of different shear forces between regions during deformation. Proteoglycans and elastic fibres in the IFM may be involved in the regulation of collagenous matrix and could enhance the lubrication of collagen bundles and elastic recoil mechanisms at this site. Together, these findings may relate to different functioning of ACL and LDET and indicate that ACL is subjected to more compressive forces, resulting in different ECM composition and arrangement, which could make the tissue more susceptible to or could protect the tissue from damage.

## Author's contribution

Y.A.K., S.T., E.L. and E.C. designed the experiments. Y.A.K. conducted experiments, evaluated the results, and prepared the manuscript. S.T., E.L. and E.C. evaluated the results and prepared the manuscript. All authors have read and approved the final submitted manuscript.

## Conflict of interest

The authors declare no conflict of interest.

## Supporting information


**Fig. S1.** Location of division.
**Fig. S2.** Negative controls.
**Fig. S3.** Additional histology scoring results.

**Table S1**. Histology scoring systems.

**Table S2**. Antibody details.
