# Supplementary material for: Variations in internal structure, composition and protein distribution between intra‐ and extra‐articular knee ligaments and tendons
Source: J Anat. 2018 Mar 2;232(6):943–55. doi: 10.1111/joa.12802 (PMC5978954; doi:10.1111/joa.12802)
Supplement: Supplementary file 1 — Fig. S1. Location of division. Fig. S2. Negative controls. Fig. S3. Additional histology scoring results. [file JOA-232-943-s001.docx]

**­Supplementary Figure 1: Location of division**

**
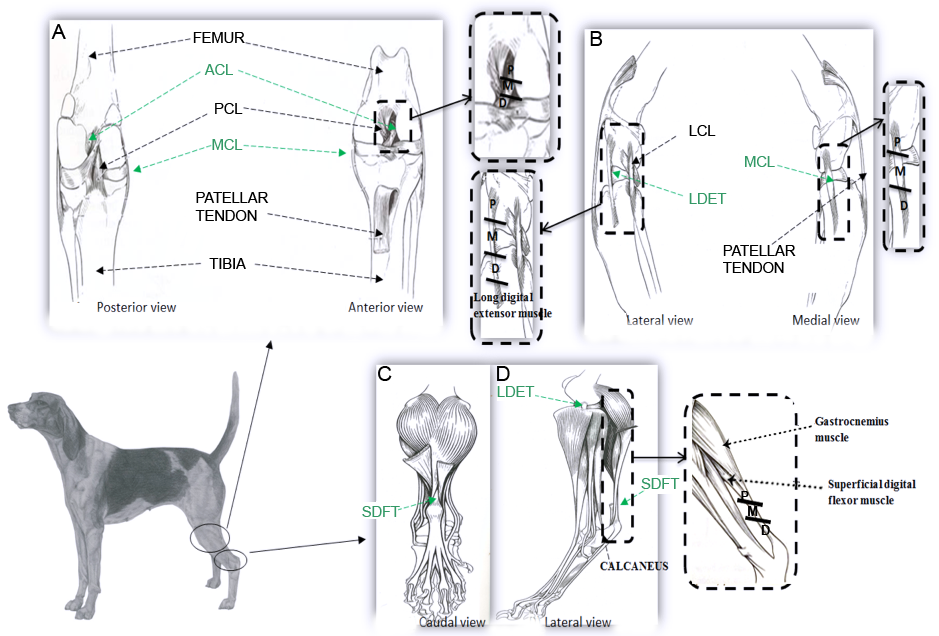
**

Anatomy of canine stifle or knee joint which is adapted from (Evans et al. 1979). Locations of tissues collected are indicated by the green arrows. Figure A and B indicate the left and right stifle joint with the position of ACL, MCL and LDET. Figure C and D indicates caudal and lateral view of pelvic limb, showing the position of LDET and SDFT. Dashed boxes indicate areas that are magnified in the subsequent images to represent the location of division into proximal, middle and distal parts of ligaments and tendons.

**Supplementary Figure 2: Negative controls**


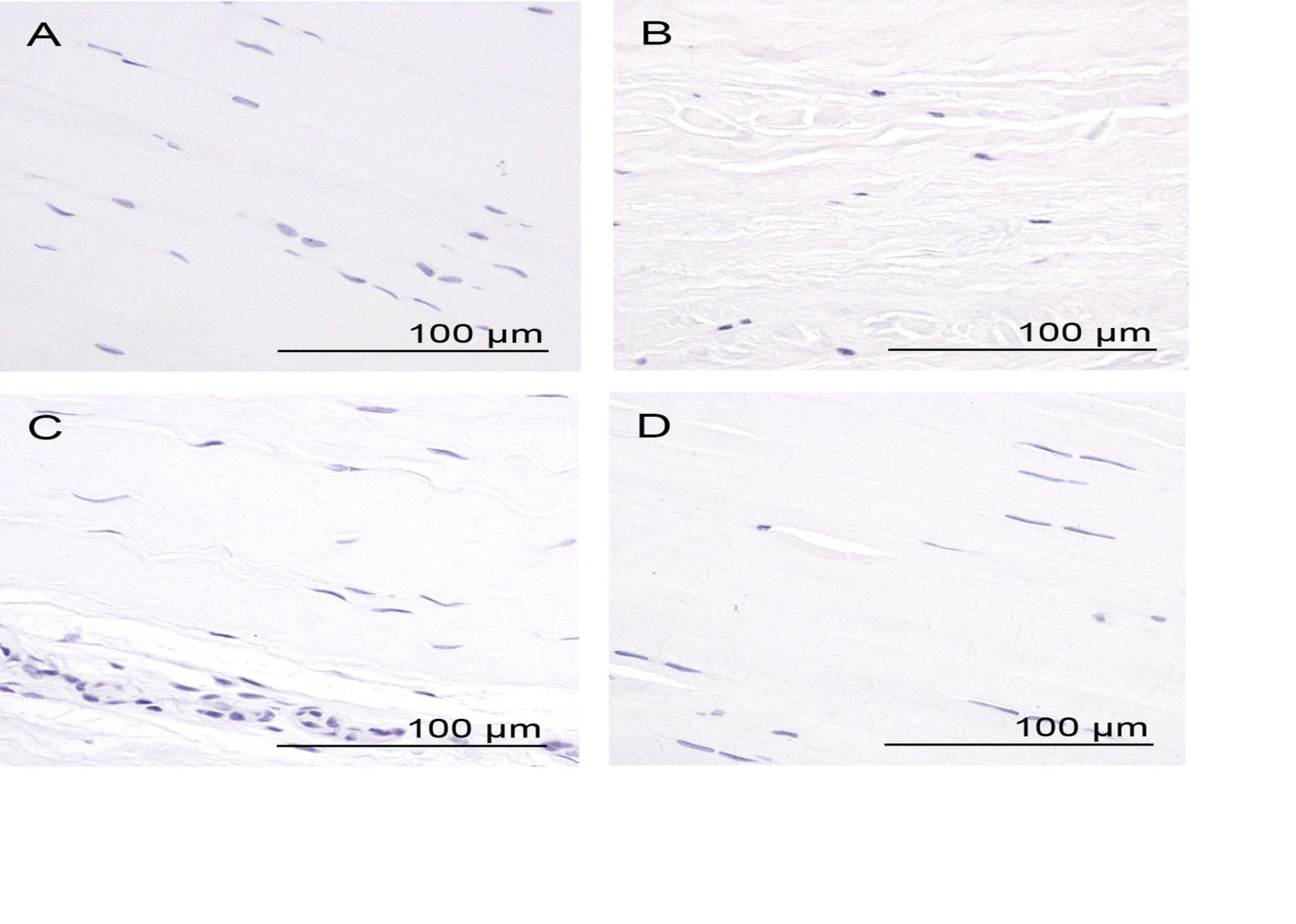


Representative immunostaining pictures of negative controls. Bar 100µm and 40X magnifications. No immunostaining was detected in the absence of primary antibody with addition of secondary goat antirabbit (A), secondary goat antimouse (B), rabbit IgG (C) and mouse IgG (D).

**Supplementary Figure 3: Additional histology scoring results**

**A**

Histological scoring for cell distribution was not significantly different between tissue types and proximal (P), middle (M) and distal (D) locations of each tissue. Values are average of scores and error bars represent SD.


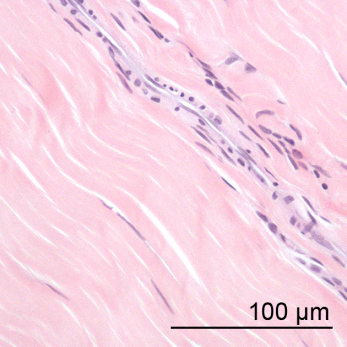
**B**

SDFT middle region

Histology scoring for vascularisation demonstrated that SDFT was statistically more vascularised compared to ACL (*P*<0.001), MCL (*P*<0.001) and LDET (*P*<0.001). No significant differences were found between different locations in each tissue. Values are average of scores and error bars represent SD.


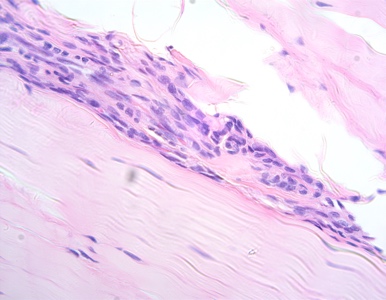

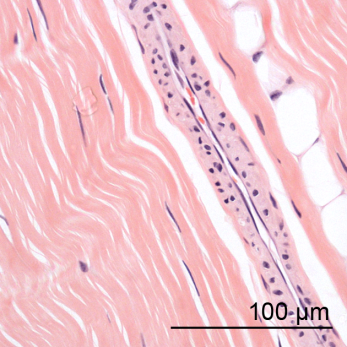
**C**

SDFT middle region

Histology scoring for inflammation demonstrated that SDFT had statistically more infiltrating cells than ACL (*P*<0.001), MCL (*P*<0.001) and LDET (*P*<0.005). No significant differences were found between different locations in each tissue. Values are average of scores and error bars represent SD.

**Reference**

Evans, H. E., G. C. Christensen and A. De La Hunta (1979). "MILLER’S (1980)“Guide to the Disection of the Dog”. 2 nd-Ed." Philadelphia, WB-Saunders.
