# Supplementary material for: Variations in internal structure, composition and protein distribution between intra‐ and extra‐articular knee ligaments and tendons
Source: J Anat. 2018 Mar 2;232(6):943–55. doi: 10.1111/joa.12802 (PMC5978954; doi:10.1111/joa.12802)
Supplement: Supplementary file 2 — Table S1. Histology scoring systems. [file JOA-232-943-s002.docx]

**Supplementary table 1: Histology scoring systems**

| **1) Extracellular matrix organization of the whole ligament or tendon Score**   - Wavy, compact and parallel arranged collagen fibre 2   (Normal collagen architecture and density)   - In part compact, in part loose or not orderly 1   (Reduced density)   - Loosely composed, not orderly 0   (Absent, minimal density)  **2) Cellular shape, distribution and alignment**   1. ***Shape***  - Spindle shape (normal) 2 - Mixed 1 - Oval to round shape 0  1. ***Distribution***  - Normal distribution of cells 1 - focal areas of elevated cell density 0   (Cell clustering or formation of cell chains)   1. ***Alignment***  - Uniaxial 2 - Areas of irregular arranged cells 1 - More than 50% of cell with no uniaxial alignment 0   **3) Vascularity of the whole ligament and/or tendon**   - hypo-vascularised 1 - hyper-vascularised (increased number 0   of smaller or larger capillaries) |
| --- |
|  |

H&E scoring sheet adapted from Stoll, John et al ([2011](#_ENREF_304)). Each parameter was numerically graded from 0-2 based on changes seen for each parameter listed and the average score between inter and intra-observers was calculated.

| **1) Distribution and location of Microfibril staining Score**   1. *Distribution*  - Normal (sparse fibres throughout) 0 - Increased 1  1. *Location*   ***Interfascicular matrix***   \|  \| >50% \| 25-50% \| 0-25% \| 0% \| \| --- \| --- \| --- \| --- \| --- \| \| - Interfascicular \|  \|  \|  \|  \| \|  \| >50% \| 25-50% \| 0-25% \| 0% \| \| - Interbundle \|  \|  \|  \|  \| \| ***Fascicular matrix*** \| >50% \| 25-50% \| 0-25% \| 0% \| \| - Substance \|  \|  \|  \|  \|     **2) Pericellular staining**   1. Pericellular deposition  - not present 0 - present 1  \|  \| >50% \| 25-50% \| 0-25% \| 0% \| \| --- \| --- \| --- \| --- \| --- \| \|  \|  \|  \|  \|  \|  1. Degree of pericellular deposition |
| --- | --- | --- | --- | --- | --- | --- | --- | --- | --- | --- | --- | --- | --- | --- | --- | --- | --- | --- | --- | --- | --- | --- | --- | --- | --- | --- | --- | --- | --- | --- | --- | --- | --- | --- | --- | --- | --- | --- | --- | --- |

Miller’s scoring sheet adapted from Smith, Clegg et al. ([2013](#_ENREF_296)). For the general distribution and presence of pericellular staining a score between 0-1 was given. For the interfascicular, interbundle, intrabundle, fascicular and pericellular staining a score from 0-3 was awarded based on the extent of staining 0%= 0, absent, 0-25%= 1, staining percent in up to 25% of tissue, 25-50%=2, staining present in 25-50 percent of tissue, >50%= 3, marked staining in above 50% of the tissue. The overall score was added up for each sample, giving a range of 0-14.

| **1) Glycosaminoglycans distribution and location**   1. ***Overall***  - normal (absent) 0 - Increased (focal or generalised) 1  1. ***location***   ***Interfascicular matrix:***   \|  \| >50% \| 25-50% \| 0-25% \| 0% \| \| --- \| --- \| --- \| --- \| --- \| \| - Interfascicular \|  \|  \|  \|  \| \|  \| >50% \| 25-50% \| 0-25% \| 0% \| \| - Interbundle \|  \|  \|  \|  \| \| ***Fascicular matrix:*** \| >50% \| 25-50% \| 0-25% \| 0% \| \| - Substance \|  \|  \|  \|  \|   **2) Cell with ‘chondroid’ change**   1. ***Cell shape***  - No change (well-spaced, spindle shaped) 0 - Oval to rounding of nuclei (halos around them) 1  \| >50% \| 25-50% \| 0-25% \| 0% \| \| --- \| --- \| --- \| --- \| \|  \|  \|  \|  \|  1. ***Location of stain with cells***  - Around the cells |
| --- | --- | --- | --- | --- | --- | --- | --- | --- | --- | --- | --- | --- | --- | --- | --- | --- | --- | --- | --- | --- | --- | --- | --- | --- | --- | --- | --- | --- | --- | --- | --- | --- | --- | --- | --- | --- | --- | --- |

Alcian-blue-PAS scoring sheet. For the general distribution and presence of pericellular staining a score between 0-1 was given. For the interfascicular, interbundle, intrabundle, fascicular and pericellular staining a score from 0-3 was awarded based on the extent of staining 0%= 0, absent, 0-25%= 1, staining percent in up to 25% of tissue, 25-50%=2, staining present in 25-50 percent of tissue, >50%= 3, marked staining in above 50% of the tissue. The overall score was added up for each sample, giving a range of 0-14.
