# Supplementary material for: Variations in internal structure, composition and protein distribution between intra‐ and extra‐articular knee ligaments and tendons
Source: J Anat. 2018 Mar 2;232(6):943–55. doi: 10.1111/joa.12802 (PMC5978954; doi:10.1111/joa.12802)
Supplement: Supplementary file 3 — Table S2. Antibody details. [file JOA-232-943-s003.docx]

**Supplementary Table 2: Antibody details**

| Primary antibody | Manufacturer | Secondary antibody |
| --- | --- | --- |
| Collagen I (1:100) | Abcam (ab292) | ZytoChemPlus (HRP) Polymer anti-Rabbit (ZUC032) |
| Collagen III (1:100) | Abcam (ab7778) | ZytoChemPlus (HRP) Polymer anti-Rabbit (ZUC032) |
| Collagen VI (1:100) | Abcam (ab6588) | ZytoChemPlus (HRP) Polymer anti-Rabbit (ZUC032) |
| Asporin (1:100) | Abcam (ab58741) | ZytoChemPlus (HRP) Polymer anti-Rabbit (ZUC032) |
| Decorin (70.6) (1:50) | Donated by B.Caterson/ C Hughes (Cardif university) | Anti-Mouse IgG (1:50)  (A4416, Sigma, UK) |
| Biglycan (PR8A4) (1:50) | Donated by B.Caterson/C.Hughes | Anti-Mouse IgG  (1:50) (A4416, Sigma, UK) |
| Keratocan (KER-1) (1:50) | Donated by B.Caterson/ C. Hughes | Anti-Mouse IgG  (1:50) (A4416, Sigma, UK) |
| Agreccan (7D1) (1:50) | Donated by B.Caterson/C. Hughes | Anti-Mouse IgG  (1:50) (A4416, Sigma, UK) |
| Versican (1:100) | Hybridoma (12C5) | Anti-Mouse IgG  (1:50) (A4416, Sigma, UK) |
| Elastin (1:100) | Abcam (ab9519) | Anti-Mouse IgG (1:500) (A11001, Invitrogen, USA) |
| Fibrillin 1 (1:50) | Donated by R. Mecham (Washington University) | Anti-Rabbit IgG (1:500) ( A11011, Invitrogen, USA) |
| Fibrillin 2 (1:50) | Donated by T. Ritty (Penn State University) | Anti-Rabbit IgG (1:500) ( A11011, Invitrogen, USA) |

Primary and secondary antibodies used in tissue immunostaining for detection of extracellular matrix macromolecules.
